# Potentially inappropriate medications in relation to length of nursing home stay among older adults

**DOI:** 10.1186/s12877-021-02639-3

**Published:** 2022-01-22

**Authors:** Eva Sönnerstam, Maria Gustafsson, Hugo Lövheim

**Affiliations:** 1 Department of Integrative Medical Biology, Umeå University, SE-901 87 Umeå, Sweden; 2 Department of Community Medicine and Rehabilitation, Umeå University, 901 87 Umeå, Sweden

**Keywords:** Older adults, Major neurocognitive disorders, Potentially inappropriate medications, Nursing homes, Length of stay

## Abstract

**Background:**

To investigate the use of potentially inappropriate medications and their relation to the length of nursing home stay among older adults.

**Methods:**

Questionnaire surveys using the Multi-Dimensional Dementia Assessment scale were sent out to all nursing homes in Västerbotten county in northern Sweden in 2007 and 2013. In total, 3186 adults (1881 from 2007 and 1305 from 2013) ≥65 years old were included and 71.8% of those had cognitive impairment. Potentially inappropriate medications were identified using drug-specific quality indicators according to Swedish National Board of Health and Welfare.

**Results:**

Potentially inappropriate medications were used by 48.0% of the 2007 study sample and by 28.4% of the 2013 study sample. The prevalence of glibenclamide use 2007 and antipsychotic drug use 2013 increased linearly (β = 0.534E^− 3^, 95% CI: 0.040E^− 3^-0.103E^− 2^, *p* = 0.034 and β = 0.155E^− 2^, 95% CI: 0.229E^− 3^-0.288E^− 2^, *p* = 0.022, respectively) with the length of nursing home stay. No significant association was found between the prevalence of propiomazine, codeine, long-acting benzodiazepines, anticholinergics, NSAIDs, tramadol or the total use of potentially inappropriate medications and the length of stay in nursing homes in 2007 or 2013. Antipsychotics were the most commonly prescribed of the drug classes investigated and used by 22.6% of the residents 2007 and by 16.0% of the residents 2013.

**Conclusions:**

These results indicate that treatment with potentially inappropriate medications is common among older adults living in nursing homes, but it seems to be related to the length of nursing home stay only to a smaller extent. Drug treatment should regularly be reviewed and followed-up among nursing home residents regardless of their length of nursing home stay, in order to prevent unnecessary adverse events.

**Supplementary Information:**

The online version contains supplementary material available at 10.1186/s12877-021-02639-3.

## Background

Older adults living in nursing homes have more comorbidities compared to their counterparts in the wider community [1] and major neurocognitive disorders are common [2]. The number of older adults with major neurocognitive disorders increases exponentially after the age of 65 and it is a common cause of being admitted to a nursing home [3, 4]. Consequently, as the number of older adults grow in society, so does the number of older adults with major neurocognitive disorders and those living in nursing homes [3].

Older adults in nursing homes and especially those with major neurocognitive disorders, are vulnerable to drugs and more prone to experience adverse drug reactions and hospital admissions [1, 3, 5]. One study found that 18.8% of acute hospital admissions were due to adverse drug reactions [6]. Drug related problems are common among nursing home residents [7] and it is found that these problems often are preventable [8]. Nevertheless, nursing home residents are often subject to polypharmacy and are prescribed more potentially inappropriate medications (PIMs) such as psychotropic drugs, compared to community-dwelling older adults [1, 9–11]. Antipsychotics and benzodiazepines are two type of psychotropic drug classes that are commonly prescribed, especially to older adults experiencing behavioural and psychological symptoms in dementia (BPSD) [12]. More specifically, antipsychotics are utilized to treat agitation, aggression, delusions and hallucinations [13], i.e. behavioural disturbances associated with major neurocognitive disorders that nursing home residents often experience [4]. Although antipsychotics are associated with severe side effects such as extrapyramidal symptoms, impaired cognition, cerebrovascular events and even increased risk of mortality [14–16], they are often used among residents without appropriate indications [1, 12]. Moreover, benzodiazepines increase the risk of fall accidents and cognitive impairment [17]. Other PIMs that are often prescribed are anticholinergic drugs which contribute to cognitive impairment and delirium [18].

Trends indicate that PIM use has increased over time among nursing home residents [19]. On the other hand, a decrease of antipsychotic drug use in recent years was found among nursing homes residents living in Norway [20] and Sweden [21–24]. A previous study has found that having a higher number of pro re nata prescriptions, i.e. prescribed drugs used as needed, is associated with length of nursing home stay above 2.1 years [25]. Moreover, it was found that having a higher number of medications prescribed is associated with the length of stay in nursing homes [26]. These findings are supported by a Belgian study conducted among older adults in nursing homes [27]. However, the association between the prevalence of PIMs and length of nursing home stay among residents has not yet been determined.

Therefore, we aimed to investigate the prevalence of PIMs and the association between PIM use and the length of stay in nursing homes among older residents using Swedish explicit quality indicators.

## Methods

### Data collection

To determine the association between the prevalence of PIM use and the length of stay in nursing homes, data were collected from two cross-sectional surveys that were sent to all residents living in nursing homes in the county of Västerbotten in northern Sweden. The same questionnaire was sent twice, in May 2007 and 2013 respectively, and included an individual assessment of the residents’ condition the preceding week. This should be filled in by the member of the staff who knew the resident best and was completed without direct involvement of the residents. Written instructions how the assessments should be carried out were included in the questionnaire and the staff were informed to contact the research team by telephone if questions arose. Moreover, a registered nurse was always instructed to complete data about the residents’ currently prescribed medications and attach a copy of the current drug list before the questionnaire was sent back. Information about when the resident was admitted to the nursing homes was also noted in the questionnaire. Results from these surveys have previously been reported [23, 24, 28–31].

### Data assessments

The Multi-Dimensional Dementia Assessment Scale (MDDAS) was used to assess the residents’ health condition [32]. This assessment scale has good inter- and intra-rater reliability [32] and measures cognition, BPSD, hearing, speech, vision, motor functions and activities of daily living (ADL).

The MDDAS covers 14 different psychological symptoms related to mental health and 25 different behavioural symptoms. Each of the item is rated on a three-point scale dependent on how often the symptoms occurs, i.e. at least once a day, once a week or never during the preceding week. The scale therefore measures a one-week prevalence of BPSD occurrence among the residents if dichotomised between never and at least once a week.

The ADL score is based on the residents’ ability to cope with dressing, eating, hygiene and bladder and bowel control. Every ADL category ranges between 1 and 5 except bowel control, which ranges between 0 and 4. Therefore, the sum of the ADL score varies from 4 to 24 where a higher score indicates a higher ADL independence.

A scale developed by Gottfries and Gottfries was utilised to measure the level of cognitive function among the residents [29, 33]. The scale consists of 27 items and ranges between 0 and 27 points. A score less than 24 indicates cognitive impairment and correlates with 90% sensitivity and 91% specificity [32] to the cut-off of 24/30 used in the Mini-Mental State Examination (MMSE) [34]. The score was further categorised into mild cognitive impairment (score 16-23), moderate cognitive impairment (score 8-15) and severe cognitive impairment (score 0-7).

### Data extraction

Older adults with an identifiable year noted on the questionnaire, indicating when the person moved in to the nursing home, were qualified for inclusion in the study. If no valid month was noted, the individual got a random value of 1-12, or 1-6 if they moved in to the nursing home the same year as the questionnaire was distributed.

Moreover, a random value of 1-30 was imputed for those residents who lacked a specified day. Thereafter, the length of nursing home stay was estimated by calculating the time difference between when the questionnaire was sent out and when the resident moved in to the nursing home. Some individuals, however, got a value < 0. Consequently, these individuals were excluded. Finally, the length of nursing home stay was truncated as complete months for an individual with a value ≥0.

### Definition of potentially inappropriate medications

Drug-specific quality indicators, listed by the Swedish National Board of Health and Welfare, were used to identify PIMs among the residents’ drug lists [16]. The following drugs or drug classes, listed as inappropriate regardless of indication, were included in the analysis: long-acting benzodiazepines, anticholinergic drugs, tramadol, propiomazine, codeine and glibenclamide. Moreover, Non-Steroidal Anti-Inflammatory Drugs (NSAIDs) and antipsychotic drugs (except lithium) were included. According to Swedish indicators correct and current indication is important in order to classify NSAIDs and antipsychotic drugs as inappropriate. This information was however not available. Because of the many side effects associated with these drugs [14] they were classified as inappropriate medications regardless of indication in accordance with another study [24].

The research team identified and grouped the drugs according to World Health Organization (WHO) Anatomical Therapeutic Index (ATC) drug classification system. PIM use was defined as having at least one of the specified drugs prescribed regardless of treatment duration. Seven drug names were introduced on the Swedish market after 2007 and were therefore only identified among the residents in 2013. Consequently, missing values arose for these substances among respondents in 2007. These missing values were recoded as “did not use” (*n* = 1881) to enable correct prevalence calculations for all identified substances. Moreover, the attached drug list only provided information about the residents’ ongoing drug treatment. Consequently, pro re nata drugs could not be included in the analysis.

### Study population

There were 3578 and 3210 older adults living in geriatric care in Västerbotten county in 2007 and 2013, respectively and the response rate was 85.8% (*n* = 3070) in 2007 and 70.5% (*n* = 2262) in 2013. Geriatric and psychogeriatric hospital wards were classified as geriatric care in 2007 but not in 2013. Respondents from these units were therefore excluded (*n* = 99) from the study population to include a homogenous sample from both years. Those younger than 65 years old or those for whom no age was registered (*n* = 278), older adults with missing values on the Gottfries scale (*n* = 569) and older adults without a complete medication list (*n* = 238) were excluded. Residents with a length of nursing home stay exceeding 5 years (*n* = 962) were also excluded to minimise a healthy survivor effect and to assure that no resident was included twice in the present study. Finally, respondents from 2007 (*n* = 1881) and 2013 (*n* = 1305) were included. Those with missing values (ADL, *n* = 52 (2007), *n* = 71 (2013) and sex, *n* = 4 (2007), *n* = 6 (2013)) were excluded from the regression analyses. An additional file, including a flowchart (Fig. A1), shows the inclusion process in more detail [see Additional file 1].

### Statistics

Dichotomous variables are reported as frequencies. Variables with normal distribution are presented as mean values with standard deviation (SD) and variables with skewed distribution are reported as median with interquartile range (IQR). The prevalence of older adults using the different PIMs, PIM classes and total PIMs were plotted against the length of nursing home stay for the individual 2007 and 2013 study samples. To investigate the association between the use of PIMs in older adults and the length of nursing home stay 2007 and 2013, separate multilinear regression analyses were fitted to and conducted for each PIM or PIM class according to classification in the indicators. Finally, a multilinear regression analysis was conducted to investigate the association between individuals with at least one of the PIMs regardless of classification, i.e. total PIMs and the length of nursing home stay. However, as the PIM groups are not independent of the total PIM analysis, of which they are subsets, and also that all single significant associations are interpreted cautiously, it was decided that a multiple testing approach would not be necessary. The prevalence of older adults using at least one of the different PIMs, PIM classes and total PIMs was entered as the dependent variable into the model. Length of stay in months was entered as an independent variable into the model and the analyses were adjusted according to sex (entered as a dichotomous variable), age, level of cognitive function, ADL and number of medications (all entered as continuous variables). For comparison purpose, separate analyses were performed to investigate the relationship between PIMs, PIM classes and total PIMs respectively and the length of nursing home stay in the 2007 and 2013 populations without adjustment for number of medications. Consequently, these analyses were only adjusted for sex, age, level of cognitive function and ADL. Moreover, a supplementary multilinear regression analysis was conducted, investigating the association between number of medications and length of nursing home stay for the 2007 and 2013 populations. Number of medications was entered as a dependent variable and length of stay in months was entered as an independent variable into the model. These analyses were adjusted for sex, age, level of cognitive function and ADL. Only complete cases (*n* = 1825 (2007) and *n* = 1228 (2013)) were included in the regression analyses. Linear regression curves were fitted to the data when the length of nursing home stay was statistically significant (*p* < 0.05). Moreover, the unstandardized β for length of stay was presented together with 95% confidence interval (CI) and the *p*-value. All statistical analyses were performed using IBM SPSS Statistics version 26.

## Results

Basic characteristics of the residents are listed in Table 1. Two thirds were women and the mean age was 84.2 years (± 6.7) and 84.9 years (± 6.9) in the 2007 and 2013 study samples, respectively. The length of nursing home stay ranged between 0 and 60 months for both study samples and 50% of the older adults had stayed for 6.0-33.0 months (IQR) in 2007 and 7.0-32.0 months (IQR) in 2013. Seven out of 10, 72.0%, of the 2007 study population and 71.5% of the 2013 study population had cognitive impairment according to Gottfries’ score. The mean ADL score was 15.8 (± 6.1) among the residents 2007 and 16.1 (± 6.0) among the 2013 study population.

**Table 1:** Basic characteristics of the study samples 2007 and 2013

	2007	2013
Total number of older adults, n	1881	1305
Women^a^, n (%)	1272 (67.6)	886 (67.9)
Age, mean ± SD	84.2 ± 6.7	84.9 ± 6.9
Severe cognitive impairment^b^, n (%)	357 (19.0)	217 (16.6)
Moderate cognitive impairment^c^, n (%)	486 (25.8)	349 (26.7)
Mild cognitive impairment^d^, n (%)	512 (27.2)	367 (28.1)
No cognitive impairment^e^, n (%)	526 (28.0)	372 (28.5)
Gottfries’ score, mean ± SD	16.4 ± 8.5	16.8 ± 8.1
ADL score (4-24)^f^, mean ± SD	15.8 ± 6.1	16.1 ± 6.0
Number of medications ± SD	7.8 ± 3.5	7.7 ± 3.6
Length of nursing home stay – number of months, IQR (Range)	6.0-33.0 (0-60)	7.0-32.0 (0-60)

*SD* Standard deviation, *ADL* Activities of daily living, *IQR* Interquartile range

^a^Missing: 2007, *n* = 4; 2013, *n* = 6

^b^Gottfries’ scale 0-7

^c^Gottfries’ scale 8-15

^d^Gottfries’ scale 16-23

^e^Gottfries’ scale 24-27

^f^Missing: 2007, *n* = 52; 2013, *n* = 71

By using explicit quality indicators to identify PIM use among the residents, we found out that the prevalence of older adults using glibenclamide in 2007 and the prevalence of older adults using antipsychotic drugs in 2013 increased linearly (β = 0.534E^− 3^, 95% CI: 0.040E^− 3^-0.103E^− 2^, *p* = 0.034 and β = 0.155E^− 2^, 95% CI: 0.229E^− 3^-0.288E^− 2^, *p* = 0.022, respectively) as the length of stay in nursing homes increased (Fig. 1.6a and 1.8b) when adjusted for age, sex, level of cognitive function, ADL and number of medications. However, no significant association was found between the prevalence of residents using long-acting benzodiazepines, anticholinergic drugs, tramadol, propiomazine, codeine, NSAIDs or the total use of PIMs and the length of stay in nursing homes for neither study population (Fig. 1.1-5, 1.7 and 1.9). Moreover, no significant association was found between the prevalence of residents using glibenclamide in 2013 or for the prevalence of residents using antipsychotic drugs in 2007 and the length of stay in nursing homes (Fig. 1.6b and 1.8a).

**Fig. 1:**
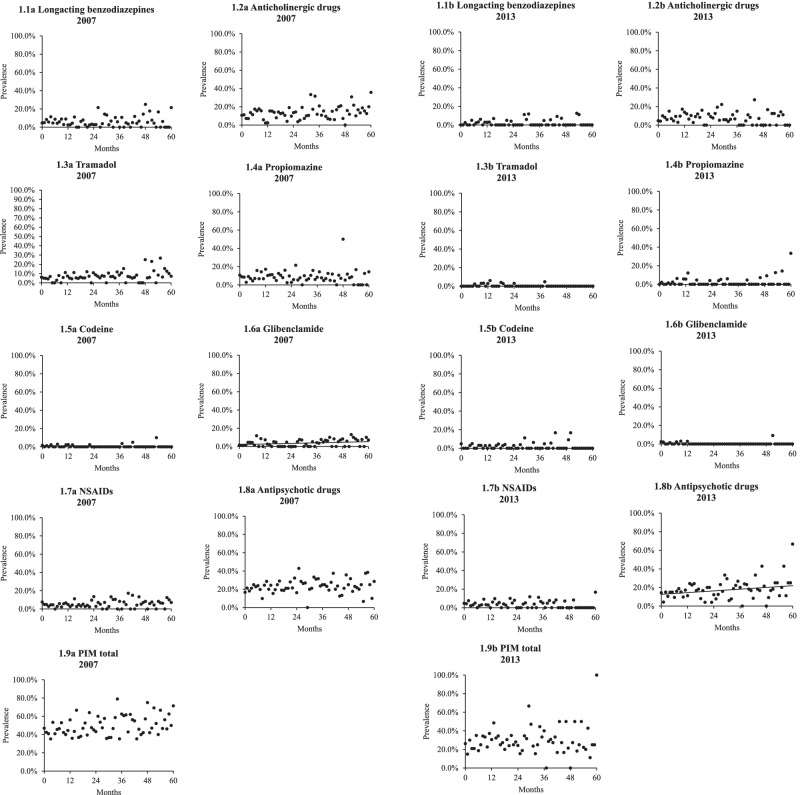
The prevalence of older adults using PIMs in relation to length of stay in months. NSAIDs: Non-Steroidal Anti-Inflammatory Drugs; PIM: Potentially inappropriate medication

An additional file provides results for supplementary analyses (Fig. A2), presenting that the association between the prevalence of tramadol use, glibenclamide use and the use of at least one PIM and the length of nursing home stay were significant in 2007 (β = 0.797E^− 3^, 95% CI: 0.121E^− 3^-0.147E^− 2^, *p* = 0.021; β = 0.636E^− 3^, 95% CI: 0.143E^− 3^-0.113E^− 2^, *p* = 0.011 and β = 0.157E^− 2^, 95% CI: 0.188E^− 3^-0.296E^− 2^, *p* = 0.026, respectively), when number of medications was removed as an independent variable from the model. Moreover, the prevalence of older adults using antipsychotic drugs was still significantly associated with length of nursing home stay in 2013 (β = 0.162E^− 2^, 95% CI: 0.281E^− 3^-0.295E^− 2^, *p* = 0.018) when the analysis only was adjusted for age, sex, level of cognitive function and ADL. Additional analyses (Fig. A3) found a significant linear association between the number of medications and length of nursing home stay among participants in 2007 (β = 0.025, 95% CI: 0.015-0.034, *p* < 0.001) [See Additional file 2].

Table 2 presents the number and frequencies of residents using PIMs. In total, 48.0% of the 2007 study population and 28.4% of the 2013 study population were prescribed at least one of the identified PIMs. The most commonly used drug class among the residents was antipsychotics where 22.6% (*n* = 426) and 16.0% (*n* = 209) of the older adults in the 2007 and 2013 study samples, respectively, were affected. Risperidone (*n* = 178, 9.5% (2007); *n* = 91, 7.0% (2013)), olanzapine (*n* = 92, 4.9% (2007); *n* = 54, 4.1% (2013)) and haloperidol (*n* = 89, 4.7% (2007); *n* = 25, 1.9% (2013)) were the most commonly prescribed substances within that drug class. Anticholinergic drugs were the second most commonly prescribed drug class and were identified among 12.7% (*n* = 238) of the residents in 2007 and among 8.0% (*n* = 105) of the residents in 2013. Hydroxyzine (*n* = 56, 3.0% (2007); *n* = 9, 0.7% (2013)), ketobemidone and antispasmodics (*n* = 36, 1.9% (2007); n = 23, 1.8% (2013)) and amitriptyline (*n* = 32, 1.7% (2007); *n* = 19, 1.5% (2013)), were the most commonly used drugs. Furthermore, propiomazine was the third most commonly prescribed drug among them in 2007, *n* = 164 (8.7%). However, the third most commonly prescribed PIM class among the 2013 study population was NSAIDs, *n* = 48 (3.7%). An additional file provides a supplementary table of all PIMs (Table A1) that were included from the Swedish quality indicators along with the frequency of residents using each PIM or PIM class [see Additional file 3].

**Table 2:** Frequency of residents using each of the identified PIM or PIM classes 2007 and 2013

	2007	2013
**Total number of participants in study sample**	**1881**	**1305**
**Long-acting benzodiazepines,** ***n*** **(%)**^**a**^	**112 (6.0)**	**23 (1.8)**
Diazepam (N05BA01), *n* (%)	15 (0.8)	6 (0.5)
Nitrazepam (N05CD02), *n* (%)	15 (0.8)	1 (< 0.1)
Flunitrazepam (N05CD03), *n* (%)	85 (4.5)	18 (1.4)
**Anticholinergic drugs, n (%)**^**a**^	**238 (12.7)**	**105 (8.0)**
*Gastrointestinal agents, anticholinergic*		
Glycopyrronium (A03AB02), *n* (%)	0	2 (0.2)
*Anticholinergic antiemetics*		
Scopolamine (A04AD01), *n* (%)	3 (0.2)	3 (0.2)
*Antiarrhythmics class 1A*		
Disopyramide (C01BA03), *n* (%)	2 (0.1)	0
*Urinary antispasmodics (excl G04BD12)*		
Tolterodine (G04BD07), *n* (%)	24 (1.3)	4 (0.3)
Solifenacin (G04BD08), *n* (%)	1 (< 0.1)	2 (0.2)
Darifenacin (G04BD10), *n* (%)	1 (< 0.1)	1 (< 0.1)
Fesoterodine (G04BD11), *n* (%)	0	2 (0.2)
*Opiates and opioids in combination with antispasmodics*		
Ketobemidone and antispasmodics (N02AG02), *n* (%)	36 (1.9)	23 (1.8)
*Anticholinergic anti-Parkinsonian drugs*		
Trihexyphenidyl (N04AA01), *n* (%)	5 (0.3)	4 (0.3)
Biperiden (N04AA02), *n* (%)	10 (0.5)	2 (0.2)
*Antipsychotic drugs*		
Levomepromazine (N05AA02), *n* (%)	21 (1.1)	13 (1.0)
Chlorprothixene (N05AF03), *n* (%)	1 (< 0.1)	0
Clozapine (N05AH02), *n* (%)	13 (0.7)	3 (0.2)
*Anxiolytics*		
Hydroxyzine (N05BB01), *n* (%)	56 (3.0)	9 (0.7)
*Antidepressants, non-selective monoamine reuptake inhibitors*		
Clomipramine (N06AA04), *n* (%)	6 (0.3)	1 (< 0.1)
Amitriptyline (N06AA09), *n* (%)	32 (1.7)	19 (1.5)
*Antihistamines*		
Clemastine (R06AA04), *n* (%)	14 (0.7)	10 (0.8)
Alimemazine (R06AD01), *n* (%)	29 (1.5)	11 (0.8)
Promethazine (R06AD02), *n* (%)	4 (0.2)	2 (0.2)
Meclozine (R06AE05), *n* (%)	0	1 (< 0.1)
**Tramadol (N02AX02),** ***n*** **(%)**	**119 (6.3)**	**10 (0.8)**
**Propiomazine (N05CM06),** ***n*** **(%)**	**164 (8.7)**	**25 (1.9)**
**Codeine,** ***n*** **(%)**	**13 (0.7)**	**23 (1.8)**
Paracetamol/codeine (N02AJ06), *n* (%)	13 (0.7)	21 (1.6)
Codeine (R05DA04), *n* (%)	0	2 (0.2)
**Glibenclamide (A10BB01),** ***n*** **(%)**	**62 (3.3)**	**7 (0.5)**
**NSAIDs (COX-inhibitors),** ***n*** **(%)**^**a**^	**97 (5.2)**	**48 (3.7)**
Indometacin (M01AB01), *n* (%)	1 (< 0.1)	0
Diclofenac (M01AB05), *n* (%)	25 (1.3)	16 (1.2)
Tenoxicam (M01AC02), *n* (%)	1 (< 0.1)	0
Ibuprofen (M01AE01), *n* (%)	10 (0.5)	8 (0.6)
Naproxen (M01AE02), *n* (%)	26 (1.4)	10 (0.8)
Ketoprofen (M01AE03), *n* (%)	33 (1.8)	15 (1.1)
Celecoxib (M01AH01), *n* (%)	1 (< 0.1)	0
Etoricoxib (M01AH05), *n* (%)	1 (< 0.1)	0
Nabumetone (M01AX01), *n* (%)	4 (0.2)	0
**Antipsychotic drugs,** ***n*** **(%)**^**a**^	**426 (22.6)**	**209 (16.0)**
Levomepromazine (N05AA02), *n* (%)	21 (1.1)	13 (1.0)
Dixyrazine (N05AB01), *n* (%)	2 (0.1)	0
Perphenazine (N05AB03), *n* (%)	6 (0.3)	2 (0.2)
Haloperidol (N05AD01), *n* (%)	89 (4.7)	25 (1.9)
Melperone (N05AD03), *n* (%)	14 (0.7)	10 (0.8)
Ziprasidone (N05AE04), *n* (%)	3 (0.2)	1 (< 0.1)
Flupenthixol (N05AF01), *n* (%)	3 (0.2)	3 (0.2)
Chlorprothixene (N05AF03), *n* (%)	1 (< 0.1)	0
Zuclopenthixol (N05AF05), *n* (%)	21 (1.1)	3 (0.2)
Clozapine (N05AH02), *n* (%)	13 (0.7)	3 (0.2)
Olanzapine (N05AH03), *n* (%)	92 (4.9)	54 (4.1)
Quetiapine (N05AH04), *n* (%)	7 (0.4)	18 (1.4)
Risperidone (N05AX08), *n* (%)	178 (9.5)	91 (7.0)
Aripiprazole (N05AX12), *n* (%)	0	1 (< 0.1)
**PIMs total,** ***n*** **(%)**^**b**^	**903 (48.0)**	**370 (28.4)**

*NSAIDs* Non-Steroidal Anti-Inflammatory Drugs, *COX-inhibitors* Cyclooxygenase inhibitors, *PIMs* Potentially inappropriate medications

^a^The frequency and prevalence differ from the sum of PIMs within the class, because some older adults used more than one PIM

^b^The frequency and prevalence differ from the sum of PIMs in total, because some older adults used more than one PIM from several PIM classes

## Discussion

This paper describes how the prevalence of PIM use is associated to the length of stay in nursing homes. We did not find any association between total PIM use and increasing length of nursing home stay in none of the study populations and the prevalence of PIMs in the study population was already high among those newly admitted to the nursing homes both in 2007 and 2013. The prevalence of PIM use among the 2007 study population is similar to another where 44.3% of the population with mild or major neurocognitive disorders had at least one PIM prescribed before admission to nursing homes [35]. It is well known that those living in nursing homes and especially those with major neurocognitive disorders have more chronic conditions, more medications and PIMs, which support the findings of the high PIM prevalence in the present study and the significant association between number of medications and length of nursing home stay which was found among the 2007 study population [1, 9, 10]. Moreover, we found that total PIM use increased linearly with an increasing length of nursing home stay in the 2007 population when number of medications was removed from the model in the comparing analysis. Previous studies have found an increased risk of having PIMs when having a higher number of medications prescribed [36, 37]. PIM use is also found to be more common among frail residents than those with better health status at admission [35]. Moreover, the risk of being prescribed one or more PIMs after transition to a nursing home was found to be more common among frail older adults [35]. The result in the present study therefore implies that PIMs are also prescribed in the community or hospital settings before admission to nursing homes and that PIM use is, just to a lesser extent, associated with the length of nursing home stay and type of accommodation. This is supported by previous studies showing no association between PIM and type of accommodation among older adults admitted to hospital [36, 38, 39]. Consequently, the high PIM prevalence might be due to a higher disease burden and higher number of medications among those that have stayed longer and just, to a lesser extent, to the length of stay. This is supported by the supplementary analyses showing a significant linear association between the number of medications and increasing length of stay in nursing homes among the 2007 study population. Moreover, the associations between PIMs and length of nursing home that were found in the comparison analyses for the 2007 study sample, may be explained by the association between length of stay and number of medications. A high proportion, 72.0 and 71.5% of the residents in 2007 and 2013 respectively, have cognitive impairment, which is similar to a previous study [2]. PIMs in general are associated with increased risks of adverse drug reactions and consequently hospital admissions among older adults with cognitive impairment [6]. The high prevalence of PIMs among nursing home residents therefore warrants concern, regardless of their length of nursing home stay.

More than one fifth of the residents 2007 used some type of antipsychotic drug which is higher compared to one study [40] but lower compared to other studies [35, 41, 42]. In 2013, the prevalence of residents using antipsychotic drugs was lower, 16.0%, and consequently lower compared to the previously mentioned studies [35, 40–42]. No association was found between the prevalence of antipsychotic drug use and length of stay in nursing homes in 2007. This indicates that a significant proportion of the residents have antipsychotic drugs prescribed before admission to the nursing homes, in line with previous findings that antipsychotic drug use is high among those admitted from hospitals to nursing homes [40]. However, in 2013 a linear association was found between the prevalence of people using antipsychotic drugs and the length of stay in nursing homes. New prescribing habits and increasing prevalence of BPSD among those that have stayed longer in nursing homes might explain this relationship. BPSD is common among older adults with major neurocognitive disorders and consequently among residents living in nursing homes [4]. This can explain the high prevalence of antipsychotic drug use which contributes to the high prevalence of total PIM use in both study populations. Even if the antipsychotic drug use has decreased, this type of drug treatment is important to highlight because of its association with increased risk of mortality and cerebrovascular events among older adults with major neurocognitive disorders [14, 43].

Additionally, more than every tenth resident in 2007 and almost every tenth resident in 2013 were prescribed some type of anticholinergic drug in the present study. This contributed to the overall PIM prevalence among the residents and is worth highlighting due to the associated side effects such as impaired memory and delirium [18]. This type of drug treatment may therefore reinforce pre-existing symptoms among a group of adults where cognitive impairment is common and acetylcholine levels are lowered [2, 16].

Glibenclamide was the second substance for which there was a significant linear association with length of nursing home stay. This was found for the 2007 population. The small increase might be due to old prescribing habits, e.g. glibenclamide was more commonly prescribed historically and consequently this drug is more common among those who have stayed longer in nursing homes in 2007 if the drug has not been deprescribed. The utilized tool to define PIM use was revised in 2017 and was the first version that included glibenclamide as a PIM. The recommendations regarding glibenclamide use were therefore different in 2007 and 2013 when the surveys were distributed, which might explain the prevalence of glibenclamide use found in 2007.

Altogether, the changes observed in PIM prevalence in 2007 and 2013 might be due to the first revision of the Indicators for evaluating the quality of older people’s drug therapy, developed by the Swedish National Board of Health and Welfare. This revision was made in 2010 [44]. Moreover, those counties which reduced the prevalence of PIMs included in the indicators by 10% among people 65 years or older, was offered a reward by the Swedish government in 2012. The decrease in prevalence might therefore be due to old prescribing habits, e.g. tramadol was more commonly prescribed 2007 compared to 2013. These trends are supported by a previous study [31]. Higher awareness of side effects and medication reviews may therefore explain the declining prescribing prevalence between the years. Nevertheless, it is important to continuously question the use of PIMs because of its associated side effects among the older adults. Adverse reactions become extra harmful among those with major neurocognitive disorders [16].

Strengths that should be mentioned are the large study populations of unselected nursing home populations, 2007 and 2013, and generally good quality of recorded data. Given the large sample size and robustness of linear regression, ordinary linear regression was assumed even if the distribution of length of nursing home stay was somewhat skewed. It should therefore be noted that the models probably are more affected by individuals with shorter length of stay. It should also be noted that no adjustment for multiple testing was made. Consequently, there is potential for increased false discovery rate. The response rate was 15% lower in 2013 than in 2007 which might be a reason for the smaller study sample in 2013. This may have contributed to the different PIM prevalence observed in 2007 and 2013. Moreover, explicit criteria were used when identifying PIMs in the present study and this is also one of the limitations, i.e. we did not have any information about diagnoses or indication of drug treatment. We also lacked information on how often the indication and drug treatment were evaluated and we did not have any information about the dose or duration of treatment. Even if this information was lacking, we chose to evaluate prevalence of antipsychotics and NSAIDs due to the serious side effects associated with this type of PIMs. Consequently, some treatment with antipsychotics and NSAIDs might have been appropriate. We also lacked information about pro re nata medications which may have affected the prevalence of long-acting benzodiazepines, tramadol and codeine that might be prescribed with this type of dosage. A healthy survivor effect might have influenced the prevalence of identified PIMs, i.e. those who have stayed longer in nursing home might be healthier than those who died before. In addition, due to having survived longer in nursing homes they may have been prescribed more medications and more PIMs.

## Conclusions

Finally, we conclude that the length of stay just had a small impact on the prevalence of potentially inappropriate medications among nursing home residents. The high prevalence of potentially inappropriate medications found in the study suggests that future interventions should question this type of drug treatment among all residents regardless of their length of nursing home stay. Special focus should be directed to antipsychotic and anticholinergic drugs in order to prevent drug related side effects among nursing home residents.

## Supplementary Information

**Additional file 1:** Flowchart study participants, Flowchart showing the inclusion criteria for study participants 2007 and 2013.

**Additional file 2:** Supplementary analyses, Supplementary graphs presenting the relation between prevalence of PIM users and length of nursing home stay when number of medications was removed from the model. The relation between mean number of medications and length of nursing home stay is also presented. 

**Additional file 3:** Supplementary PIM table, Supplementary table listing all PIMs that were included from the Swedish quality indicators and the frequency of residents using each PIM or PIM class.

## Data Availability

Data can be obtained from the corresponding author on reasonable request.

## Abbreviations

ADL: Activities of daily living
ATC: Anatomical Therapeutic Index drug classification system
BPSD: Behavioural and psychological symptoms in dementia
CI: Confidence interval
IBM: International Business Machines Corporation
IQR: Interquartile range
MDDAS: Multi-Dimensional Dementia Assessment Scale
MMSE: Mini-Mental State Examination
NSAIDs: Non-Steroidal Anti-Inflammatory Drugs
PIMs: Potentially inappropriate medications
SD: Standard deviation
SPSS: Statistical Package for Social Science
WHO: World Health Organization
