# Supplementary material for: Potentially inappropriate medications in relation to length of nursing home stay among older adults
Source: BMC Geriatr. 2022 Jan 22;22:70. doi: 10.1186/s12877-021-02639-3 (PMC8783464; doi:10.1186/s12877-021-02639-3)
Supplement: Supplementary file 1 — Additional file 1: Flowchart study participants, Flowchart showing the inclusion criteria for study participants 2007 and 2013. [file 12877_2021_2639_MOESM1_ESM.pdf]

Potentially inappropriate medications in relation to length of nursing home stay among older adults

Additional file 1 – Flowchart study participants

BMC Geriatrics

Eva Sönnerstam[1], Maria Gustafsson[1], Hugo Lövheim[2]

[1] Department of Integrative Medical Biology, Umeå University, 901 87 Umeå, Sweden

[2] Department of Community Medicine and Rehabilitation, Umeå University, 901 87 Umeå, Sweden

Corresponding Author:

Eva Sönnerstam, Department of Integrative Medical Biology, Umeå University, SE-901 87 Umeå, Sweden

Phone: +46 739725674

E-mail: eva.sonnerstam@umu.se

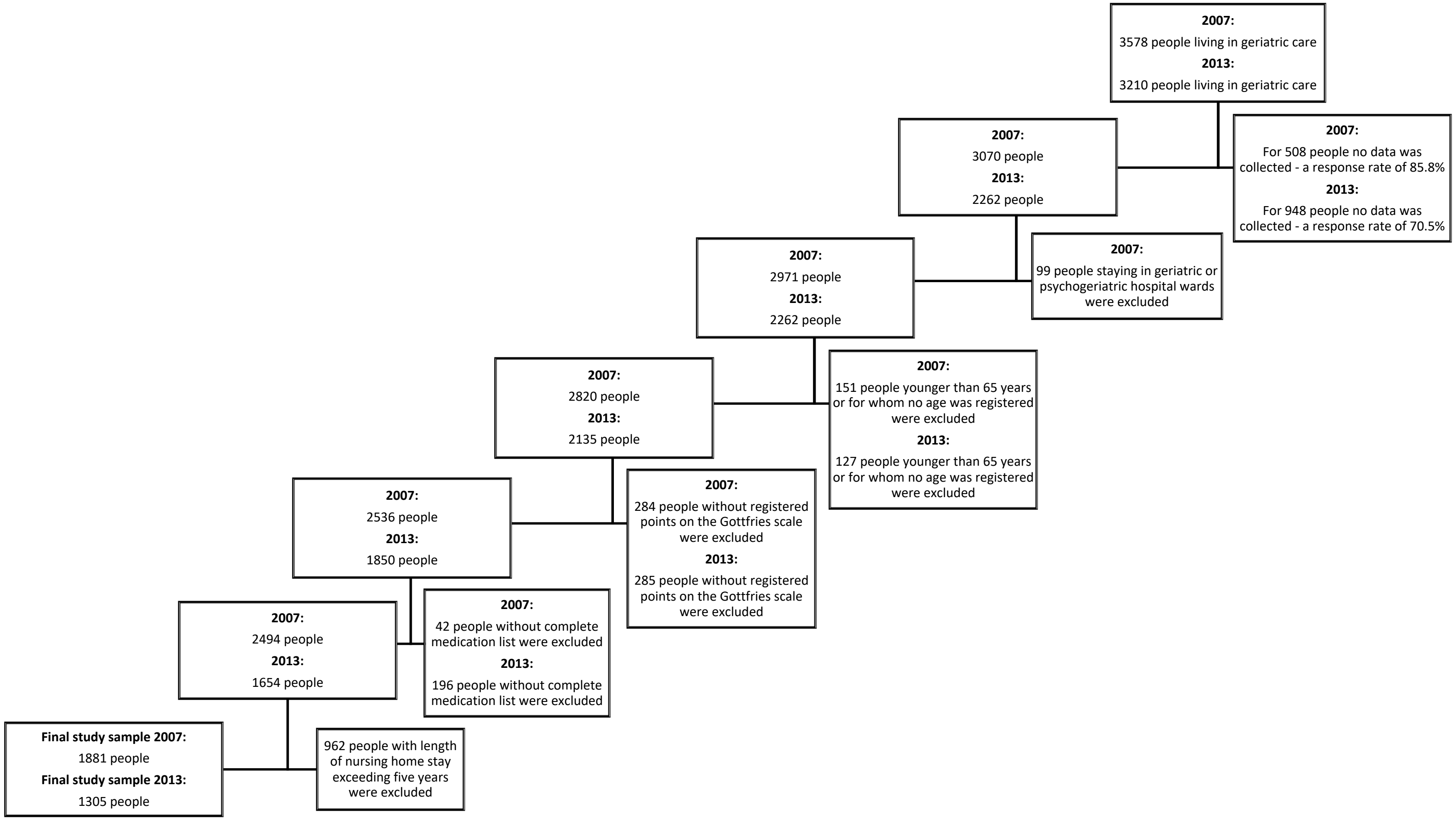

Figure A1. Flow chart showing the inclusion of participants 2007 and 2013.
