# Supplementary material for: Potentially inappropriate medications in relation to length of nursing home stay among older adults
Source: BMC Geriatr. 2022 Jan 22;22:70. doi: 10.1186/s12877-021-02639-3 (PMC8783464; doi:10.1186/s12877-021-02639-3)

Potentially inappropriate medications in relation to length of nursing home stay among older adults

Additional file 2 – Supplementary analyses

BMC Geriatrics

Eva Sönnerstam[1], Maria Gustafsson[1], Hugo Lövheim[2]

[1] Department of Integrative Medical Biology, Umeå University, 901 87 Umeå, Sweden

[2] Department of Community Medicine and Rehabilitation, Umeå University, 901 87 Umeå, Sweden

Corresponding Author:

Eva Sönnerstam, Department of Integrative Medical Biology, Umeå University, SE-901 87 Umeå, Sweden

Phone: +46 739725674

E-mail: eva.sonnerstam@umu.se

Figure A2. The following figures (fig. A2.1-A2.9) present the prevalence of PIM users in relation to length of nursing home stay for the individual 2007 (a) and 2013 (b) samples. The analyses are adjusted for age, sex, level of cognitive function and ADL. Unstandardized β together with 95% CI and p-value are presented when a significant association was found.

A2.1a Longacting benzodiazepines 2007

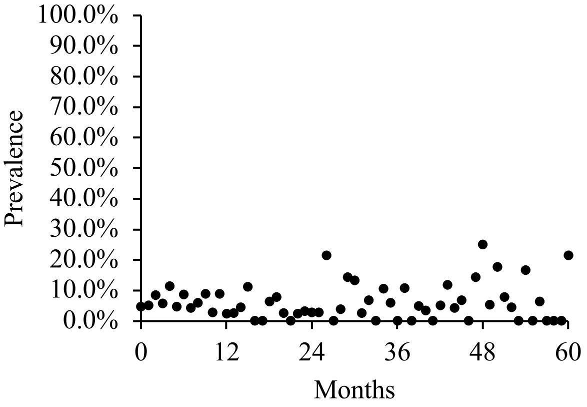

A2.1b Longacting benzodiazepines 2013

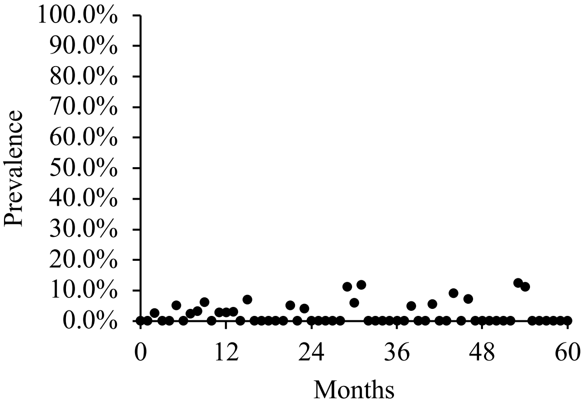

A2.2a Anticholinergic drugs 2007

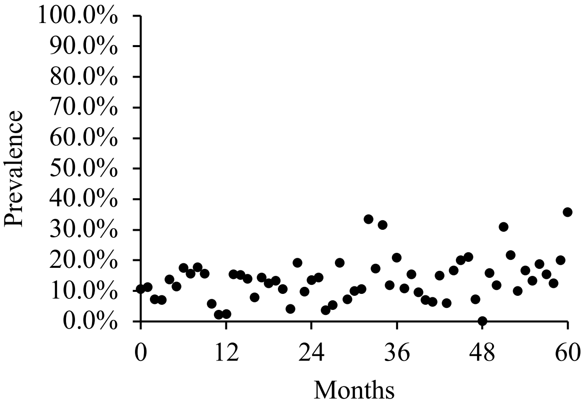

A2.2b Anticholinergic drugs 2013

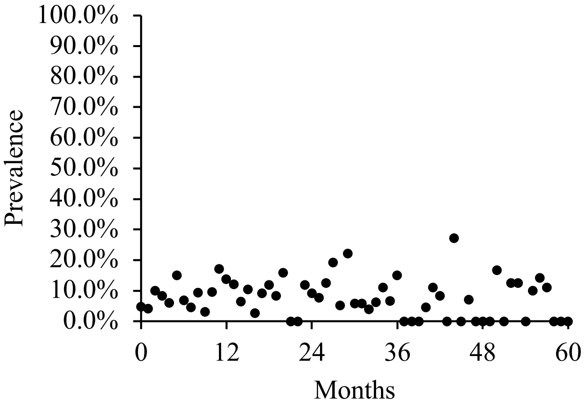

A2.3a Tramadol 2007

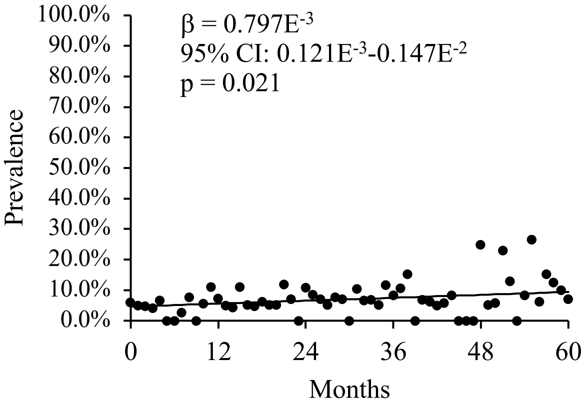

A2.3b Tramadol 2013

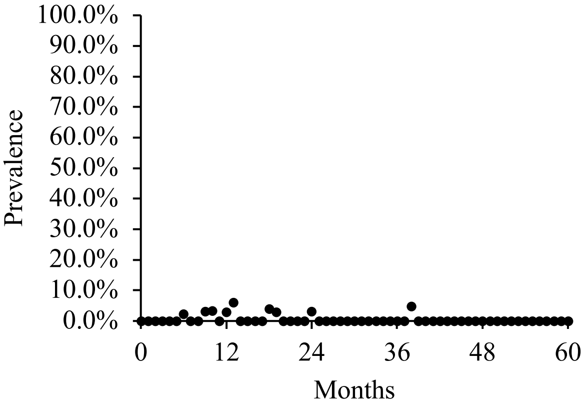

A2.4a Propiomazine
2007

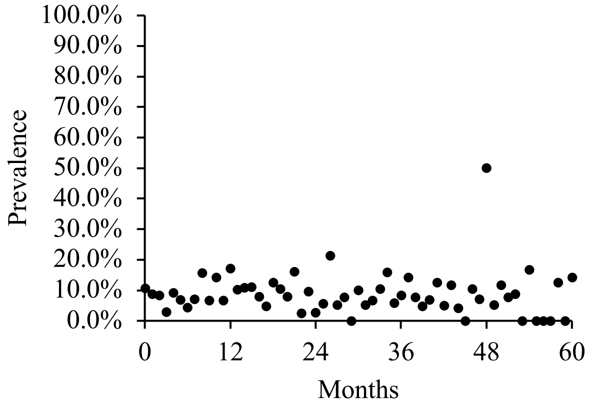

A2.4b Propiomazine
2013

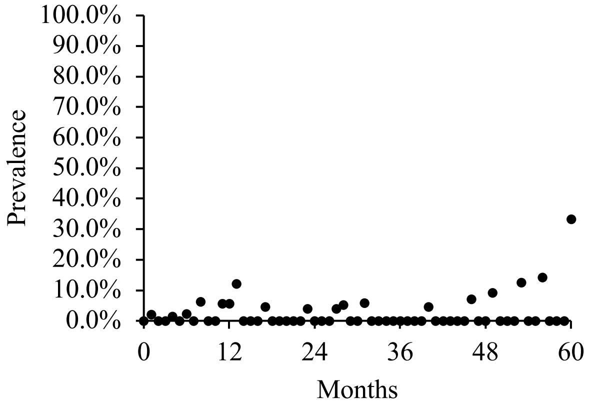

A2.5a Codeine
2007

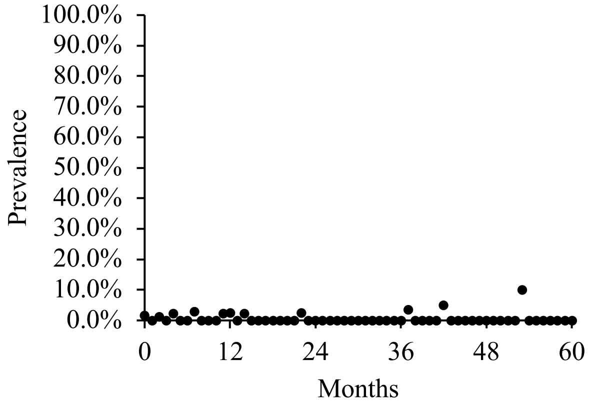

A2.5b Codeine
2013

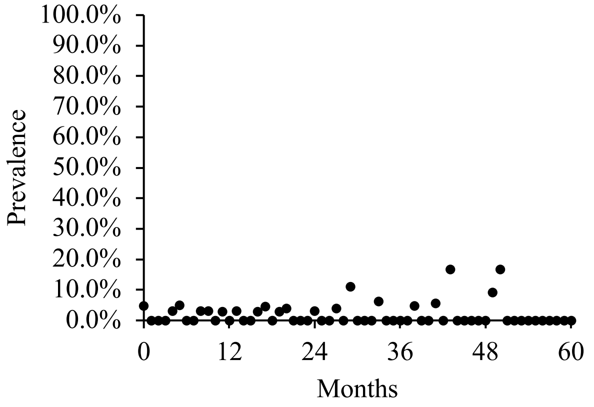

A2.6a Glibenclamide
2007

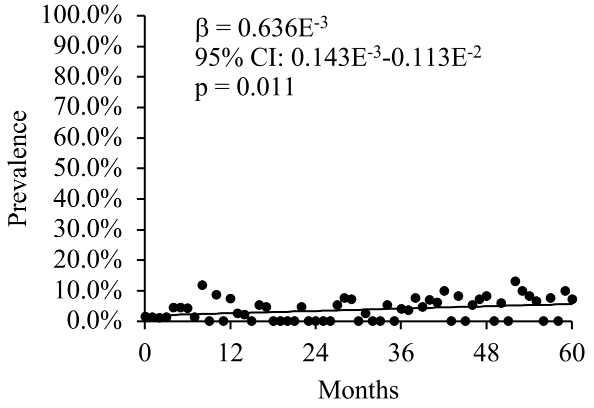

A2.6b Glibenclamide
2013

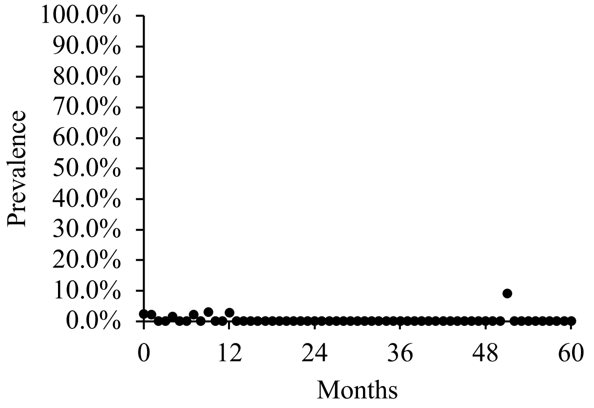

A2.7a NSAIDs
2007

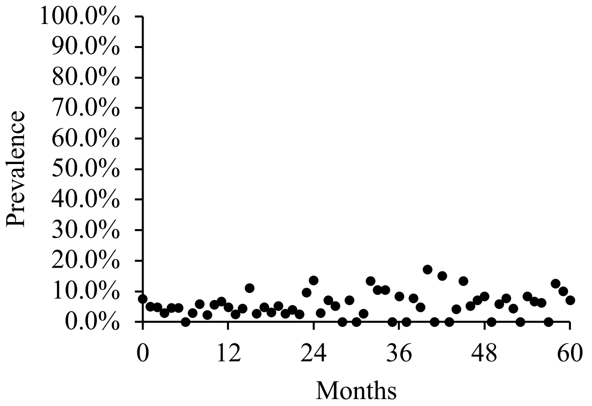

A2.7b NSAIDs
2013

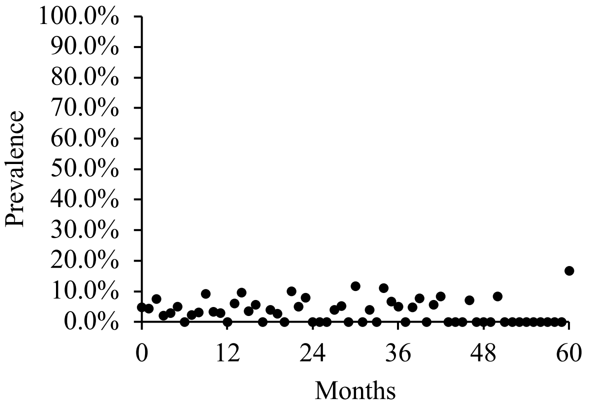

A2.8a Antipsychotic drugs
2007

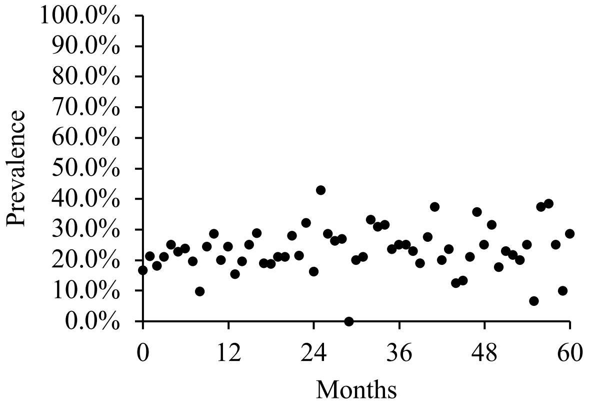

A2.8b Antipsychotic drugs
2013

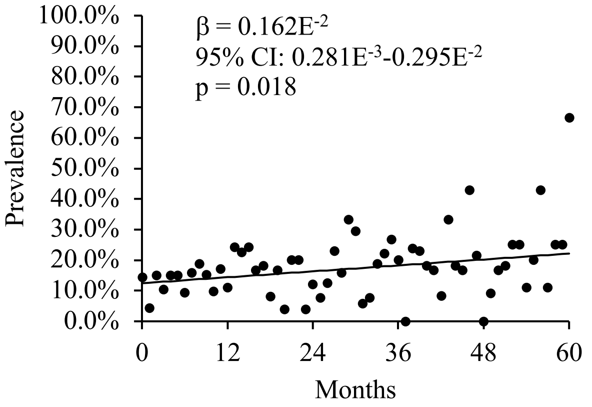

A2.9a PIM total
2007

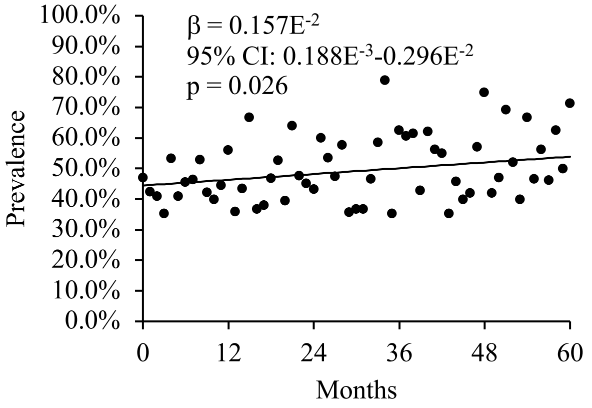

A2.9b PIM total
2013

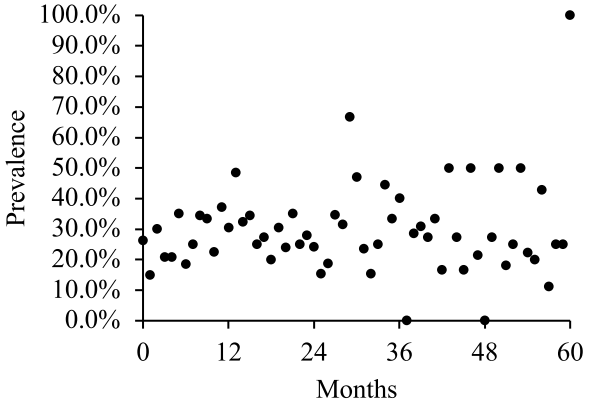

Figure A3. The following figures (fig. A3.1) present the mean number of medications in relation to length of nursing home stay for the individual 2007 (a) and 2013 (b) samples. The analyses are adjusted for age, sex, level of cognitive function and ADL. Unstandardized β together with 95% CI and p-value are presented when a significant association was found.

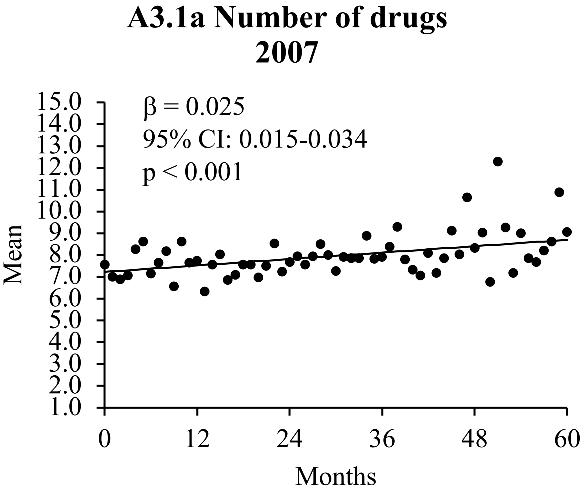

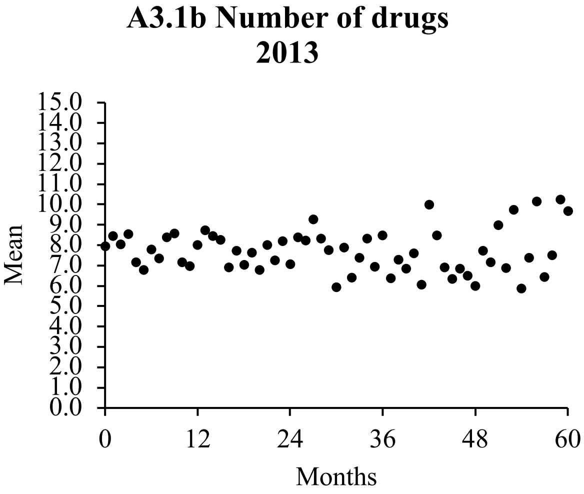

Supplement: Supplementary file 2 — Additional file 2: Supplementary analyses, Supplementary graphs presenting the relation between prevalence of PIM users and length of nursing home stay when number of medications was removed from the model. The relation between mean number of medications and length of nursing home stay is also presented. [file 12877_2021_2639_MOESM2_ESM.pdf]
