# Supplementary material for: Potentially inappropriate medications in relation to length of nursing home stay among older adults
Source: BMC Geriatr. 2022 Jan 22;22:70. doi: 10.1186/s12877-021-02639-3 (PMC8783464; doi:10.1186/s12877-021-02639-3)
Supplement: Supplementary file 3 — Additional file 3: Supplementary PIM table, Supplementary table listing all PIMs that were included from the Swedish quality indicators and the frequency of residents using each PIM or PIM class. [file 12877_2021_2639_MOESM3_ESM.pdf]

Potentially inappropriate medications in relation to length of nursing home stay among older adults

Additional file 3 – Supplementary PIM table

BMC Geriatrics

Eva Sönnerstam[1], Maria Gustafsson[1], Hugo Lövheim[2]

[1] Department of Integrative Medical Biology, Umeå University, 901 87 Umeå, Sweden

[2] Department of Community Medicine and Rehabilitation, Umeå University, 901 87 Umeå, Sweden

Corresponding Author:

Eva Sönnerstam, Department of Integrative Medical Biology, Umeå University, SE-901 87 Umeå, Sweden

Phone: +46 739725674

E-mail: eva.sonnerstam@umu.se

Table A1. Supplementary table listing all PIMs that were included from the Swedish quality indicators and the frequency of residents using each PIM or PIM class 2007 and 2013.

	**2007**	**2013**
**Total number of participants in study sample**	**1,881**	**1,305**
**Long-acting benzodiazepines, *n* (%)***	**112 (6.0)**	**23 (1.8)**
Diazepam (N05BA01), *n* (%)	15 (0.8)	6 (0.5)
Nitrazepam (N05CD02), *n* (%)	15 (0.8)	1 (<0.1)
Flunitrazepam (N05CD03), *n* (%)	85 (4.5)	18 (1.4)
**Anticholinergic drugs, n (%)***	**238 (12.7)**	**105 (8.0)**
Gastrointestinal agents, anticholinergic		
Glycopyrronium (A03AB02), *n* (%)	0	2 (0.2)
Atropine (A03BA01)	0	0
Hyoscyamine (A03BA03)	0	0
Butylscopolamine (A03BB01)	0	0
Methylscopolamine (A03BB03)	0	0
Anticholinergic antiemetics		
Scopolamine (A04AD01), *n* (%)	3 (0.2)	3 (0.2)
Antiarrhythmics class 1A		
Disopyramide (C01BA03), *n* (%)	2 (0.1)	0
Urinary antispasmodics (excl G04BD12)		
Oxybutynin (G04BD04)	0	0
Tolterodine (G04BD07), *n* (%)	24 (1.3)	4 (0.3)
Solifenacin (G04BD08), *n* (%)	1 (<0.1)	2 (0.2)
Darifenacin (G04BD10), *n* (%)	1 (<0.1)	1 (<0.1)
Fesoterodine (G04BD11), *n* (%)	0	2 (0.2)
Muscle relaxing agents, other centrally acting		
Orphenadrine (M03BC01)	0	0
Orphenadrine, combinations (M03BC51)	0	0
Opiates and opioids in combination with antispasmodics		
Morphine and antispasmodics (N02AG01)	0	0
Ketobemidone and antispasmodics (N02AG02), *n* (%)	36 (1.9)	23 (1.8)
Hydromorphone and antispasmodics (N02AG04)	0	0
Anticholinergic anti-Parkinsonian drugs		
Trihexyphenidyl (N04AA01), *n* (%)	5 (0.3)	4 (0.3)
Biperiden (N04AA02), *n* (%)	10 (0.5)	2 (0.2)
Antipsychotic drugs		
Levomepromazine (N05AA02), *n* (%)	21 (1.1)	13 (1.0
Prochlorperazine (N05AB04)	0	0
Chlorprothixene (N05AF03), *n* (%)	1 (<0.1)	0
Clozapine (N05AH02), *n* (%)	13 (0.7)	3 (0.2)
Anxiolytics		
Hydroxyzine (N05BB01), *n* (%)	56 (3.0)	9 (0.7)
Antidepressants, non-selective monoamine reuptake inhibitors		
Clomipramine (N06AA04), *n* (%)	6 (0.3)	1 (<0.1)
Amitriptyline (N06AA09), *n* (%)	32 (1.7)	19 (1.5)
Nortriptyline (N06AA10)	0	0
Maprotiline (N06AA21)	0	0
Antihistamines		
Dimenhydrinate (R06AA02)	0	0
Clemastine (R06AA04), *n* (%)	14 (0.7)	10 (0.8)
Dexchlorpheniramine (R06AB02)	0	0
Chlorphenamine (R06AB04)	0	0
Alimemazine (R06AD01), *n* (%)	29 (1.5)	11 (0.8)

Promethazine (R06AD02), *n* (%)	4 (0.2)	2 (0.2)
Tiethylperazine (R06AD03)	0	0
Meclozine (R06AE05), *n* (%)	0	1 (<0.1)
Cyproheptadine (R06AX02)	0	0
**Tramadol (N02AX02), *n* (%)**	**119 (6.3)**	**10 (0.8)**
**Propiomazine (N05CM06), *n* (%)**	**164 (8.7)**	**25 (1.9)**
**Codeine, *n* (%)**	**13 (0.7)**	**23 (1.8)**
Paracetamol/codeine (N02AJ06), *n* (%)	13 (0.7)	21 (1.6)
Codeine and other non-opioid analgesics (N02AJ09)	0	0
Codeine (R05DA04), *n* (%)	0	2 (0.2)
**Glibenclamide (A10BB01), *n* (%)**	**62 (3.3)**	**7 (0.5)**
**NSAIDs (COX-inhibitors), *n* (%)***	**97 (5.2)**	**48 (3.7)**
Indometacin (M01AB01), *n* (%)	1 (<0.1)	0
Sulindac (M01AB02)	0	0
Diclofenac (M01AB05), *n* (%)	25 (1.3)	16 (1.2)
Ketorolac (M01AB15)	0	0
Aceclofenac (M01AB16)	0	0
Diclofenac, combinations (M01AB55)	0	0
Piroxicam (M01AC01)	0	0
Tenoxicam (M01AC02), *n* (%)	1 (<0.1)	0
Lornoxicam (M01AC05)	0	0
Meloxicam (M01AC06)	0	0
Ibuprofen (M01AE01), *n* (%)	10 (0.5)	8 (0.6)
Naproxen (M01AE02), *n* (%)	26 (1.4)	10 (0.8)
Ketoprofen (M01AE03), *n* (%)	33 (1.8)	15 (1.1)
Flurbiprofen (M01AE09)	0	0
Dexibuprofen (M01AE14)	0	0
Dexketoprofen (M01AE17)	0	0
Naproxen and esomeprazole (M01AE52)	0	0
Celecoxib (M01AH01), *n* (%)	1 (<0.1)	0
Valdecoxib (M01AH03)	0	0
Parecoxib (M01AH04)	0	0
Etoricoxib (M01AH05), *n* (%)	1 (<0.1)	0
Lumiracoxib (M01AH06)	0	0
Nabumetone (M01AX01), *n* (%)	4 (0.2)	0
Bensydamine (M01AX07)	0	0
**Antipsychotic drugs, *n* (%)***	**426 (22.6)**	**209 (16.0)**
Chlorpromazine (N05AA01)	0	0
Levomepromazine (N05AA02), *n* (%)	21 (1.1)	13 (1.0)
Dixyrazine (N05AB01), *n* (%)	2 (0.1)	0
Fluphenazine (N05AB02)	0	0
Perphenazine (N05AB03), *n* (%)	6 (0.3)	2 (0.2)
Prochlorperazine (N05AB04)	0	0
Haloperidol (N05AD01), *n* (%)	89 (4.7)	25 (1.9)
Melperone (N05AD03), *n* (%)	14 (0.7)	10 (0.8)
Droperidol (N05AD08)	0	0
Sertindole (N05AE03)	0	0
Ziprasidone (N05AE04), *n* (%)	3 (0.2)	1 (<0.1)
Lurasidone (N05AE05)	0	0
Flupenthixol (N05AF01), *n* (%)	3 (0.2)	3 (0.2)
Chlorprothixene (N05AF03), *n* (%)	1 (<0.1)	0
Zuclopenthixol (N05AF05), *n* (%)	21 (1.1)	3 (0.2)
Loxapine (N05AH01)	0	0
Clozapine (N05AH02), *n* (%)	13 (0.7)	3 (0.2)
Olanzapine (N05AH03), *n* (%)	92 (4.9)	54 (4.1)
Quetiapine (N05AH04), *n* (%)	7 (0.4)	18 (1.4)
Asenapine (N05AH05)	0	0

Risperidone (N05AX08), *n* (%)	178 (9.5)	91 (7.0)
Aripiprazole (N05AX12), *n* (%)	0	1 (<0.1)
Peliperidon (N05AX13)	0	0
Cariprazine (N05AX15)	0	0
Brexpiprazol (N05AX16)	0	0
**PIMs total, *n* (%)**[**]	**903 (48.0)**	**370 (28.4)**

NSAIDs = Non-Steroidal Anti-Inflammatory Drugs, COX-inhibitors = Cyclooxygenase inhibitors, PIMs = Potentially inappropriate medications.

[*]The frequency and prevalence differ from the sum of PIMs within the class, because some older adults used more than one PIM.

[**]The frequency and prevalence differ from the sum of PIMs in total, because some older adults used more than one PIM from several PIM classes.
